# The Effects of Viruses on Insulin Sensitivity and Blood–Brain Barrier Function

**DOI:** 10.3390/ijms24032377

**Published:** 2023-01-25

**Authors:** Jacob Raber, Elizabeth M. Rhea, William A. Banks

**Affiliations:** 1 Departments of Behavioral Neuroscience, Neurology and Radiation Medicine; Affiliate Scientist, Division of Neuroscience, ONPRC, Oregon Health & Science University, Portland, OR 97239, USA; 2 Geriatric Research Education and Clinical Center, Veterans Affairs Puget Sound Health Care System, Seattle, WA 98108, USA; 3 Department of Medicine, University of Washington, Seattle, WA 98108, USA

**Keywords:** viruses, insulin resistance, blood–brain barrier

## Abstract

In this review manuscript, we discuss the effects of select common viruses on insulin sensitivity and blood–brain barrier (BBB) function and the potential overlapping and distinct mechanisms involved in these effects. More specifically, we discuss the effects of human immunodeficiency virus (HIV), herpes, hepatitis, influenza, respiratory syncytial virus (RSV), and SARS-CoV-2 viruses on insulin sensitivity and BBB function and the proposed underlying mechanisms. These viruses differ in their ability to be transported across the BBB, disrupt the BBB, and/or alter the function of the BBB. For RSV and SARS-CoV-2, diabetes increases the risk of infection with the virus, in addition to viral infection increasing the risk for development of diabetes. For HIV and hepatitis C and E, enhanced TNF-a levels play a role in the detrimental effects. The winter of 2022–2023 has been labeled as a tridemic as influenza, RSV, and COVID-19 are all of concern during this flu season. There is an ongoing discussion about whether combined viral exposures of influenza, RSV, and COVID-19 have additive, synergistic, or interference effects. Therefore, increased efforts are warranted to determine how combined viral exposures affect insulin sensitivity and BBB function.

## 1. Introduction

The recent SARS-CoV-2 pandemic has highlighted diabetes mellitus (DM) and other features of the metabolic syndrome as risk factors both for the acquisition of COVID-19 and for having more severe COVID-19 symptoms. However, this is not the first virus-related condition that has a relation with DM. Indeed, a recurring theme among the common viruses has been a connection with DM in general and in particular insulin receptor resistance, although the nature of those connections is diverse.

For example, the insulin receptor is implicated in viral responses. The insulin receptor shapes adaptive immune function through modulating T cell metabolism [1]. While the insulin receptor on T cells is not critical under basal conditions, activating conditions require the insulin receptor for inflammatory cytokine production, effector differentiation, proliferation, and potentially migration/recruitment to target organs. In a mouse model of severe influenza infection, lack of the insulin receptor in T cells diminishes their response, making these mice more susceptible to infection [1]. Specifically, T cells lacking the insulin receptor show reduced antigen-specific proliferation and compromised production of pro-inflammatory cytokines. This could potentially explain why individuals with insulin resistance pre-existing obesity and/or diabetes are often at an increased susceptibility to developing severe viral infections.

Furthermore, these viruses tend to have effects on brain functions and affect blood–brain barrier functions, sometimes similar to those altered BBB functions found in DM. The blood–brain barrier (BBB) can be affected by neurotropic viral infection, affecting the permeability and inflammatory immune response of the BBB [2]. Viral proteins can also affect the BBB endothelial barrier and the immune response of endothelial cells. In addition to endothelial cells, other cells of the neurovascular unit including pericytes and astrocytes can also be affected by viruses [2]. This raises the possibility that the viral effects on the brain could be mediated through effects on the BBB or that viral infection could exacerbate the effects of DM on the BBB.

In this review, we discuss the effects of human immunodeficiency virus (HIV), herpes, hepatitis, influenza, respiratory syncytial virus (RSV), and SARS-CoV-2 viruses on insulin sensitivity and BBB function and the proposed underlying mechanisms.

## 2. Viruses, Insulin Sensitivity, and BBB Function

### 2.1. HIV, Insulin Sensitivity, and BBB Function 

The diagnosis of acquired immune deficiency syndrome (AIDS) was originally based on the presence of opportunistic infections in patients who had no obvious reason for having such infections [3]. Symptoms were nonspecific and did not include hyperglycemia or DM. AIDS was a wasting disease with short life expectancy and so the opportunity to develop DM was limited. As effective treatments became available and life expectancy increased, AIDS became associated with hyperglycemia, insulin resistance, metabolic syndrome, and lipodystrophy [4,5]. However, most of these associations are thought to be iatrogenic, resulting from the medications used to treat AIDS and its accompanying conditions. Nevertheless, there is a literature that suggests that the natural history of untreated HIV-1 infection is associated with an increase in DM, dyslipidemia, and insulin resistance [6,7]. Risk factors for HIV-1-related DM include an increased viral load, low CD3 count, and a longer duration of AIDS.

**Figure 1:**
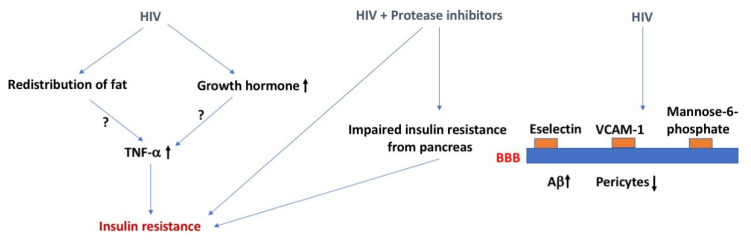
HIV, insulin resistance, and the BBB. The effects of HIV on insulin resistance might involve increases in TNF-a levels induced by redistribution of fat or enhanced growth hormone levels. HIV combined with the use of protease inhibitors as treatment can cause insulin resistance. Effects of HIV on the BBB might lead to an increase in brain Aβ levels and a decrease in pericytes. The arrows in bold indicate the direction of the effects. For more details, see text.

Several mechanisms have been proposed to explain the increase in DM with AIDS [6,7] (Figure 1). For example, the redistribution of fat could lead to increased secretion of tumor necrosis factor-α (TNF-α), resulting in insulin resistance secondary to inflammation. Dual infection with HIV-1 and hepatitis C (see also the section about hepatitis below) virus results in an increase in TNF-α and steatosis which together induce insulin resistance. AIDS is associated with a deficiency in growth hormone, a counterregulatory hormone to insulin, although it should be noted that growth hormone excess is more typically associated with DM. Case studies have recorded the onset of DM type I in patients with HAART therapy. The assumption is that the recovery of the immune system with highly active antiretroviral therapy (HAART) allows the expression of the autoimmune conditions leading to DM type I.

The strongest association between AIDS and DM, however, occurs in those treated with protease inhibitors, a common treatment for AIDS. These drugs induce dyslipidemia, lipodystrophy, and insulin resistance at muscle and adipose tissue [8]. These drugs also induce an impaired release of insulin from the pancreas [8]. Proposed mechanisms include interference with glucose transporter (GLUT)-4 activity and interference with the activities of cellular retinoic acid-binding protein type I/peroxisome proliferator-activated receptor, resulting in adipose tissue inflammation, free fatty acid release, and insulin resistance. Nucleoside reverse transcriptase inhibitors, another type of treatment for AIDS, especially stavudine, are also associated with lipodystrophy, mitochondrial damage, and insulin resistance [9]. However, DM induced by these drugs is much rarer and occurs only after prolonged use. The integrases have appeared as a risk factor for diabetes mellitus [10]. However, a study investigating AIDS patients who were switched to integrases found only a transient rise in glucose and no increase in insulin resistance [11]. Indeed, that study suggested that integrases could have a protective effect against insulin resistance.

HIV-1 crosses the BBB early in the course of the disease [12], both within infected immune cells through increased expression of e-selectin and vascular cell adhesion molecule 1 (VCAM-1) [13] and as free virus using the mannose-6 phosphate receptor [14]. The BBB is altered in many ways in patients with AIDS, including being disrupted, having increased immune cell trafficking, and altered P-glycoprotein (P-gp) activity [15,16]. The disruption of the BBB by HIV-1 and by DM are both thought to be mediated by loss of pericytes [17,18]. This raises the possibility that DM and HIV-1 infection could act synergistically in their damage of the BBB [19]. Pericytes also enhance the inflammation-induced increase in HIV-1 transcytosis [20]. HIV-1 infection also stimulates the blood-to-brain transfer of amyloid β peptide (Aβ) through a mechanism dependent on secretion of extracellular vesicles [21]. These data suggest HIV-1 infection can lead to long-lasting neurological effects due to direct changes at the BBB.

### 2.2. Herpes Virus, Insulin Sensitivity, and BBB Function 

In humans, herpes viruses are the most prevalent viruses. There are eight herpes viruses: herpes simplex virus (HSV) 1, HSV 2, varicella-zoster virus (VZV), Epstein–Barr virus (EBV), cytomegalovirus (CMV), human herpesviruses (HHV) 6, HHV 7, and HHV 8. All eight viruses result in lifelong latent infections.

**Figure 2:**
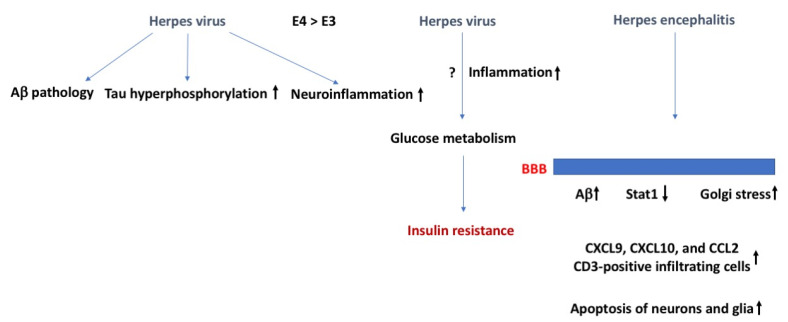
Herpes virus, insulin resistance, and the BBB. Herpes virus can induce AD-related neuropathology, including Aβ pathology, tau hyperphosphorylation, and neuroinflammation. Herpes virus might negatively affect glucose metabolism and cause insulin resistance because of increased inflammation. As there is more viral replication in E4 than E3 carriers, these effects are expected to be more pronounced in E4 than E3 carriers. In case of Herpes encephalitis, effects on the BBB might result in enhanced Aβ levels and enhanced Golgi stress and an increase in CD3-positive infiltrating cells and apoptosis of neurons and glia. The arrows in bold indicate the direction of the effects. For more details, see text.

With regard to insulin sensitivity, all herpes viruses may impair glucose metabolism and increase the risk of developing DM type II [22,23,24], a risk factor for developing Alzheimer’s disease (AD). HSV-2 and CMV are associated with increased incidence of (pre)diabetes in people with normal glucose tolerance at baseline and independent of other risk factors. The underlying mechanisms are not clear (Figure 2). Inflammation might be involved, as HSV-2 and CMV cause chronic infections and in this way might influence insulin function. At supra physiological levels, insulin can induce reactivation of inactive herpes simplex thymidine kinase gene [25]. However, insulin-like growth factor 3, which is induced following HSV-1 infection of the cornea, seems protective against Herpes Stromal Keratitis [26].

Reactivation of latent herpes virus has been hypothesized to trigger Alzheimer’s disease (AD) [27,28,29,30]. This connection was initially mainly hypothesized based on the spreading of AD pathology and viruses in brain. Herpes viruses enhance AD pathology, including intracellular and extracellular production of the amyloid precursor protein (APP), Aβ, and insoluble amyloid plaque pathology, tau hyperphosphorylation, and neuroinflammation. These AD-like characteristics might develop quickly. In cultured brain tissue, within three days after HSV-1 infection, Aβ plaques, hyperphosphorylated tau, and neuroinflammation are detected [31]. These effects are more pronounced in those carrying the genetic risk factor of AD apolipoprotein E4 [32]. The more pronounced effects in E4 carriers might relate to enhanced reactivation and the fact that for many viruses, including herpes viruses, viral replication is higher in E4 than non-E4 carriers [33].

Although often enhanced AD pathology due to herpes viruses is considered problematic, Aβ can actually inhibit HSV-1 replication and viral entry [34]. Aβ has sequence homology with HSV-1 glycoprotein B, binds HSV-1 and HHV-6 surface glycoproteins, and Aβ aggregates can trap herpes viral particles. This also raises the question of how new therapeutic strategies to remove Aβ from brain [35] might affect the susceptibility of AD patients to Herpes viral (re)activation.

Herpes simplex encephalitis is often caused by HSV-1 and involves retrograde axonal transport and reactivation of herpes simplex viruses in the olfactory bulb, trigeminal ganglia, and other tissues [36]. The permeability of the BBB increases following HSV-1 infection [37,38] and involves reduced Stat1, which is important for interferon signaling [39], Golgi stress, and downregulation of the Golgi-associated protein GM130 in endothelial cells [40]. This in turn causes brain tissue injury following influx of leukocytes and other immune mediators. HSV-1 is also proposed to cause brain injury independent of an altered BBB function. Viral activation might cause intracranial inflammation involving microglia and the chemokines CXCL9, CXCL10, and CCL2, and CD3-positive infiltrating cells [41]. HSV-1 and CMV viral replication and viral proteins also result in apoptosis of neurons and glia [42]. Individual differences in BBB function due to age or genetic factors likely play a role in determining effects of HSV-1 on the brain [43].

### 2.3. Hepatitis, Insulin Sensitivity, and BBB Function

Viral hepatitis is a common condition that has been around for a long time [44]. Over the years, hepatitis A, B, C, D, and E genotypes were identified (for a review, see [45]). Hepatitis C is associated with insulin resistance and DM [46,47], both type I [48,49] and type II [50,51]. Insulin resistance typically happens first and is not dependent on being obese or diabetic [52]. The degree of insulin resistance depends on the hepatitis C genotype, with increased insulin resistance in genotypes 1 and 4 than 2 and 3 [53,54]. This relationship seen with hepatitis C is not seen with hepatitis B. Perhaps related to this, while cognitive impairments, anxiety, and fatigue have been reported in both hepatitis B and C patients, increased hepatitis C, but not B, infection is seen in patients with dementia [55].

**Figure 3:**
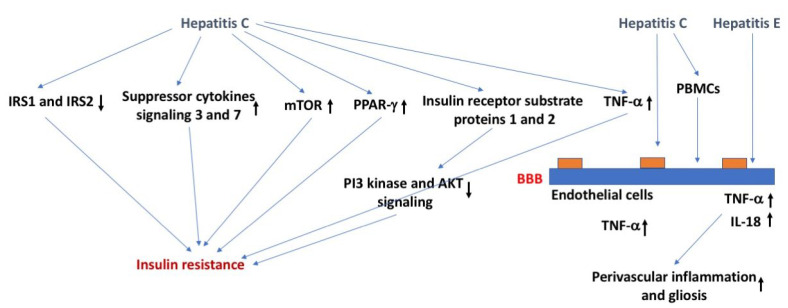
Hepatitis, insulin resistance, and the BBB. Hepatitis C might cause insulin resistance via distinct pathways. Effects of Hepatitis C on the BBB might result in enhanced TNF-α levels. These effects might involve effects of PBMCs on the BBB. Hepatitis E might cause perivascular inflammation and gliosis by increasing brain levels of TNF-α and IL-18. The arrows in bold indicate the direction of the effects. For more details, see text.

Mechanisms underlying the relation between viral hepatitis and insulin function might involve ubiquitination and downregulation of insulin receptor substrate protein 1 and 2, which are required for insulin signaling and affected in insulin resistance, through upregulation of the suppressor of cytokine signaling 3 or 7, activating the mammalian target of rapamycin, or downregulation of peroxisome proliferator-activated receptor gamma [56] (Figure 3). Viral hepatitis can also induce insulin resistance by altered phosphorylation of insulin receptor substrate protein 1 and 2, leading to their detachment from the insulin receptor, reduced phosphatidylinositol-4,5-biphosphate 3-kinase and Akt signaling, and ultimately their proteasomal degradation [57]. As indicated earlier, viral hepatitis often causes an increase in TNF-α, which can negatively affect insulin function through phosphorylation or increased levels of soluble TNF-α receptors that are often seen in patients with chronic hepatitis C [58]. Viral hepatitis might also affect insulin function by downregulating GLUT-2 and -4 [59]. In addition, viral hepatitis can affect insulin function and increase gluconeogenesis via upregulating the activity of the FOXY forkhead family of transcription factors 1 and 3 in the nucleus, increasing protein phosphatase 2A levels [60] or upregulating histone deacetylase 9 and deacetylation of forkhead box protein O1 (FOXO1) [61]. As might be expected based on all these data, antiviral treatment of patients with chronic hepatitis C improves insulin function in both diabetic and nondiabetic patients [62].

With regard to the BBB, hepatitis C can cross the BBB via endothelial cells which contain all the receptors known to act as viral attachment proteins for hepatitis C [63,64]. Hepatitis C might also enter the BBB through a Trojan horse mechanism involving infected peripheral blood mononuclear cells [65]. Importantly, the depressive symptoms, sleep, and chronic fatigue, which can affect cognitive performance, and cognitive function that might be affected directly are not fully restored following peripheral viral clearance. Following peripheral viral clearance, cognitive performance seems more improved than depressive symptoms [66]. The *APOE* genotype seems important in modulating hepatitis, with E4 carriers being relatively protected [67].

The hepatitis E virus can cross the BBB in a TNF-α independent fashion and produce a productive infection in brain endothelial cells, increasing brain TNF-α and interleukin 18 (IL-18) levels that are associated with perivascular inflammation and gliosis [68]. Interestingly, the time course of exosome-like quasi enveloped hepatitis E virions and nonenveloped hepatitis E virus was comparable [68]. Whether the remaining hepatitis virus genotypes can cross or impact the BBB remain to be determined.

### 2.4. Influenza Virus, Insulin Sensitivity, and BBB Function

There are four main types of influenza viruses: A, B, C, D. Types A and B cause seasonal epidemics of disease in people, predominantly in the winter in the United States. Influenza A viruses are the only ones known to cause flu pandemics and are named by the hemagglutinin (H) and neuraminidase (N) proteins expressed on the viral surface, such as the H1N1 pandemic from 2009. Influenza C viruses generally cause mild illness and are not related to human epidemics and influenza D viruses primarily infect cattle. Influenza viruses are negatively sensed but are separated into multiple single strands of RNA, allowing for segment reassortment to create new strains of the virus, making it difficult to design vaccines against them.

It has been proposed that influenza viruses may play a role in the etiopathogenesis of DM type I [69]. Numerous reports have linked the 2009 H1N1 pandemic to pancreatitis and the development of DM type I [69]. Influenza viruses can infect, replicate, and damage pancreatic human and mouse islets [69]. While infection in healthy mice did not cause long-term diabetes, there could be enhanced effects due to influenza strain, occurrence of repeated infections, and extent of pancreatic damage. During the final lethal stage of influenza viral infection in mice, dysregulated glucose and fatty acid metabolism and decreased TCA cycle activity was observed [70]. At 3 days post infection, liver insulin sensitivity was impaired and a tendency towards glucose intolerance was observed, particularly reflected in reduced glucose uptake.

**Figure 4:**
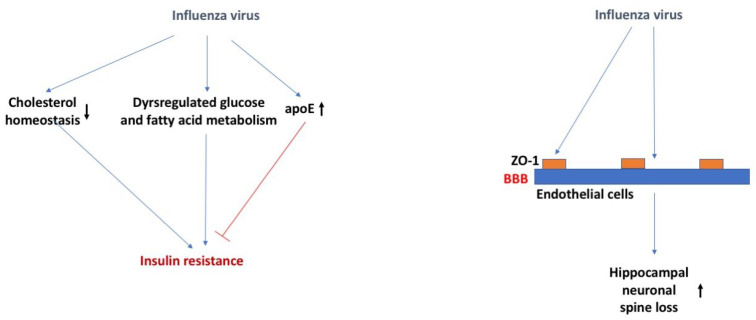
Influenza virus, insulin resistance, and the BBB. Influenza virus might cause insulin resistance by affecting cholesterol homeostasis, dysregulating glucose and fatty acid metabolism, and increasing ApoE levels. Influenza virus might cause hippocampal neuronal spine loss by affecting ZO-1 on endothelial cells of the BBB. The arrows in bold indicate the direction of the effects. For more details, see text.

Loss of ApoE in mice substantially increases the susceptibility to influenza viral infection, potentially related to the impaired cell cholesterol homeostasis allowing for enhanced viral attachment [71] (Figure 4). Influenza virus infected murine bone marrow-derived macrophages (BMDMs) had increased ApoE protein expression only when pre-treated with recombinant ApoE2 or ApoE3, but not ApoE4 [72]. Further studies showed recombinant ApoE3 prevented the influenza viral infection induced M1 polarization of BMDMs and inflammatory response. Therefore, it is possible that there is a role for the *APOE* genotype in the infection of the influenza virus. This role might not be virus specific. As indicated earlier, for many viruses, including herpes viruses, viral replication is higher in E4 than non-E4 carriers [33].

The majority of the infections in the 2009 H1N1 pandemic were pediatric. This extended to a number of neurological complications present in this population (<16 years old), including encephalopathy, despite a lack of virus detected in the CSF [73]. Pre-clinical studies show murine infection with the influenza virus results in neuronal spine loss in the hippocampus at 30 days post infection and is associated with impairments in learning, with full recovery occurring 120 days post infection [74]. Other studies have shown that cognitive impairments can occur as early as 7 days post infection and it is associated with increased neuroinflammation and altered hippocampal neuronal morphology [75].

Infection of baby chickens [76] or adult mice [74] with the influenza virus showed that the virus was able to disrupt the BBB, similar to other viruses [77], as measured by Evan’s blue extravasation, 48 h post infection or 8 days post infection, respectively. Localization of the tight junction protein ZO-1 was also disrupted at this time point and expression co-localized with viral antigen presence [76].

### 2.5. Respiratory Syncytial Virus (RSV), Insulin Sensitivity, and BBB Function 

Respiratory syncytial virus (RSV) is a common and very contagious respiratory virus that causes mild cold-like symptoms and can be severe in infants and older adults. Severe infections can include bronchiolitis and pneumonia. Additionally, RSV can make chronic health problems such as asthma and congestive heart failure worse. While the genome size of RSV is similar to the influenza virus, it contains a single, unsegmented strand of negatively sensed RNA. The surface proteins that allow for infection contain a fusion (F) protein and an attachment glycoprotein (G) that help to differentiate it from other common respiratory viruses.

There is relatively little known about how RSV may lead to insulin resistance beyond inflammatory-induced autoimmunity. Without data showing altered insulin sensitivity during or immediately following infection, it is not clear whether RSV changes insulin sensitivity immediately or whether insulin sensitivity is only impacted at a much later time due to inflammatory responses and the development of DM type I. Nevertheless, epidemiological studies postulate that RSV infection increases the relative risk for developing DM type I, suggesting RSV infection can be related to peaks of DM type I incidence [78,79,80].

Alternatively, DM type I increases the risk of RSV-positive acute respiratory illness infection with an odds ratio of 9.82 in older adults [81]. The same has been suggested for this population in children less than 5 years old by measuring length of hospital stay as a gauge for infection severity [82]. Therefore, not only can RSV infection potentially increase the risk for the development of DM type I but DM increases the risk for severity of RSV infection.

**Figure 5:**
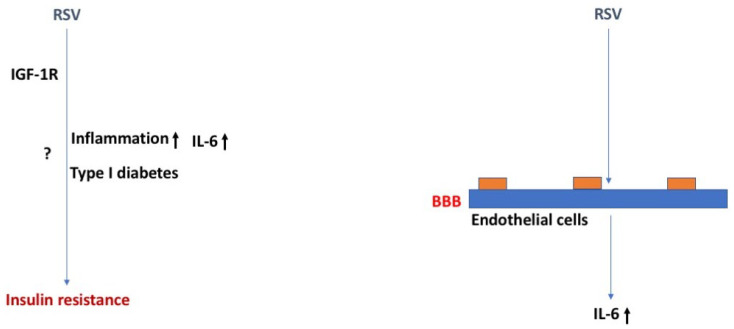
RSR, insulin resistance, and the BBB. RSV might induce insulin resistance via the IGF-1R and involve inflammation, especially enhanced IL-6 levels, and type I diabetes. The arrows in bold indicate the direction of the effects. For more details, see text.

Human adipocytes can be infected with RSV in vitro and results in over a six-fold increased IL-6 production [83], potentially contributing to the enhanced severity of this viral infection in obese populations (Figure 5). Adipocyte production of IL-6 modifies insulin sensitivity by interfering with intracellular insulin signaling pathways [84]. Furthermore, microRNA analysis performed on the blood from actively infected infants compared to controls suggests the insulin signaling pathway is significantly impacted by RSV infection, amongst other inflammatory pathways [85].

Currently, there are no vaccines against RSV and there is no treatment for the infection. However, it was recently discovered that treatment with a common diabetes agent, liraglutide—a glucagon-like peptide 1 (GLP-1) receptor agonist with anti-inflammatory properties, could reduce inflammation and the CD4^+^ T cell response in mice infected with RSV [86]. Serum insulin and glucose levels were not different in RSV-infected mice 6 days post infection. These data support a link between insulin signaling and RSV immune response. Additionally, it has been shown that the RSV F protein can interact with insulin-like growth factor 1 receptor (IGF-1R), one of many cell surface receptors facilitating host cell infection by aiding in the translocation of proteins to the cell membrane necessary for RSV internalization [87].

Similar to the other viral infections reviewed here, there is an increased risk of neurological complications in severe RSV infection requiring intensive care, including seizures, encephalopathy, and abnormal neurological examination [88,89]. CSF analysis of severely infected children presenting with seizures shows RSV can enter the CSF and IL-6 levels are highly abundant [90]. Human RSV (hRSV)-infected mice and rats display impaired learning one month after infection and reduced hippocampal LTP, with evidence for viral presence by measurement of the nucleoprotein N gene of hRSV in the brain by 3 days post infection and translocation to different regions of the CNS by 7 days post infection [91]. In addition, this group further showed that RSV entry into the brain could occur through a hematogenous pathway. Preventing CNS viral infiltration by treating with an anti-CD49d antibody, prevented the learning impairments. Follow-up studies revealed hRSV infection increases the permeability of the murine BBB measured by Evan’s blue extravasation and increases the number of CNS immune cell infiltration by 3 days post infection [77]. Endothelial cells, neurons, microglia, and astrocytes were shown to be infected with the virus in mice [77].

### 2.6. Coxsackievirus B, Insulin Sensitivity, and BBB Function

Coxsackievirus B1-6, classified as enterovirus B viruses, are the enteroviruses most associated with type I diabetes [92]. The pathways involved in Coxsackievirus B-induced autoimmunity against islets in the pancreas might involve molecular mimicry between the enteroviral protein 2C and glutamice acid decarboxylase [93], inflammation involving bystander activation of autoreactive T cells [93], prior infection in the enteric mucosa of the gut [94,95], persistence viral presence in the skeletal muscle, heart, and brain [96], an altered T cell response [97], and dysregulation of microRNAs in the pancreas [98]. Acute infection of the islets might involve enhanced IFN-*α* and impaired glucose-induced insulin secretion [99]. Chronic infections are likely involved as well. Loss of unconventional prefoldin RPB5 interactor (URI), estrogen receptor nuclear translocation leading to DNA methyltransferase 1 (DNMT1) expression, and subsequent Pdx1 promoter hypermethylation and silencing might be involved in the detrimental effects of Coxsackievirus B on insulin sensitivity [100]. Endoplasmic reticulum stress and the unfolded protein response might be involved as well [101].

Coxsackievirus B has also detrimental effects on the brain. It is frequently detected in patients with aseptic meningitis [102]. It can pass the BBB and enter the brain [103]. The effects of Coxsackievirus B on the BBB increases the permeability of the BBB by upregulating the expression of matrix metalloproteinase 9 via downregulating miRNA 1303 and degradation of junctional complexes, including Claudin4, Claudin5, VE-Cadherin, and ZO-1 [104]. In cultures of primary neurons, neutralizing serum was not able to prevent neuronal viral infection, suggesting the involvement of trans-synaptic neuronal viral transmission [105].

### 2.7. Viral Insulin/IGF-like Peptides (VILPs) and Insulin Sensitivity

Viral insulin-IGF like peptides, which show high sequence homology to IGF-1 and IGF-2, are encoded by DNA double-stranded Iridoviridae [106,107]. In humans, they have been detected in the fecal virome [108] and blood. There are single-chain and double-chain lymphocystic disease virus-1 (LCDV-1) VILPs. Single-chain VILPs have a high affinity for the IGF1R, can antagonize human IGF-1 signaling, inhibit IGF-1-induced cell proliferation and the growth hormone/IGF-1-induced growth of mice, without altering insulin signaling [106]. Both single-chain and double-chain VILPs have a low affinity for the insulin receptor. As potent and full IGF-1R agonists [109], they might affect the onset of diabetes. Some VILPs stimulate glucose uptake in white adipose tissue by increasing expression of the glucose transporter 4 [110].

### 2.8. Severe Acute Respiratory Syndrome Coronavirus 2 (SARS-CoV-2), Insulin Sensitivity, and BBB Function (Figure 6)

**Figure 6:**
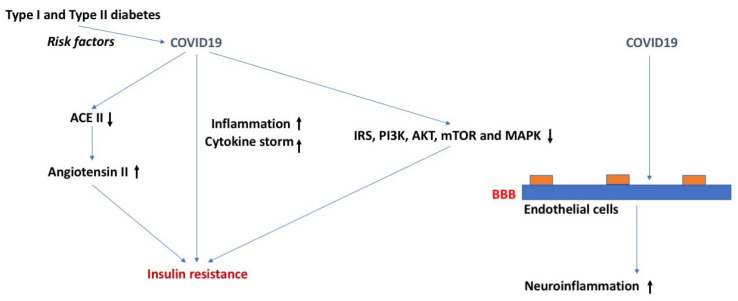
COVID-19, insulin resistance, and the BBB. Type I and type II diabetes are risk factor for COVID-19. COVID-19 might cause insulin resistance via downregulating ACE II and upregulating angiotensin II levels, increased inflammation involving a cytokine storm, or via IRS, PI3K, AKT, mTOR, and MAPK. COVID-19 might induce neuroinflammation through effects on the BBB. The arrows in bold indicate the direction of the effects. For more details, see text.

COVID-19 is another highly contagious respiratory syndrome similar to the flu and RSV. However, the SARS-CoV-2 virus contains about a two-fold greater genome than that of influenza and RSV. Additionally, SARS-CoV-2 is positively sensed, allowing the RNA to be directly translated into protein inside the host cell. Among the risk factors for either becoming infected with SARS-CoV-2 or having a worse outcome from infection are DM, obesity, and metabolic syndrome (Figure 6). For example, the risk for admission to the intensive care unit, needing mechanical ventilation, developing acute respiratory distress syndrome (ARDS), or dying because of COVID-19 is increased in those with metabolic syndrome [111]. Furthermore, the risk for developing ARDS increased as the number of metabolic syndrome features increased. In another study, those with evidence of insulin resistance, such as hypertriglyceridemia and elevated glucose, had more severe COVID-19 symptoms and an increased death rate [112].

Having COVID-19 increases the risk of developing DM. In 1902 patients with COVID-19 [113], 4% who were previously not diabetic developed DM, 27% had pre-existing DM, and another 4% developed new-onset DM. The death rate among these new-onset diabetics of 17% is exceptionally high. About half of these new-onset diabetics were still classified as diabetic a year later. Montefusco et al. [114] found new-onset DM in about 12% of COVID-19 patients and another 18.5% had transient hyperglycemia. Thus, COVID-19 has been associated with transient hyperglycemia and with new-onset DM that can be either short term or persistent [113,114].

Most of the COVID-19-related new-onset DM appears to be caused by insulin resistance rather than insulin insufficiency [114,115]. Montefusco et al. found elevated insulin and C-peptide levels not only in diabetics, but also in euglycemic COVID-19 and post-COVID patients [114]. Another group found markers of insulin resistance in non-diabetic patients that had recovered from COVID-19 3–6 months earlier [116]. These studies show that COVID-19 is not only associated with the development of insulin resistant DM, but also with its subclinical form of euglycemic hyperinsulinemia, and that these conditions may persist after recovery from acute COVID-19.

COVID-19 morbidity and mortality is also increased in those with DM type I [117]. Because this population tends to be much younger, symptoms tend to be less dramatic than those seen in the older DM type II population. However, when adjusted for age and other relevant variables, the odds ratios of dying from COVID-19 are even higher in type I than in type II diabetics [118]. A hallmark of DM type I is the development of diabetic ketoacidosis (DKA), a life-threatening event that requires emergent treatment with insulin. Pediatric patients require 18% more insulin for the treatment of their DKA if they have COVID-19 [119]. This is strong evidence that COVID-19 is associated with insulin resistance in this population as well.

Since DKA occurs when insulin activity is exceedingly low, it would be expected that DKA would be occurring more commonly during the pandemic if COVID-19 does indeed result in insulin resistance. Indeed, dramatic increases in the incidence of DKA in children with DM type I have been reported in Israel and Poland [120,121].

SARS-CoV-2 could cause DM and insulin resistance through several mechanisms. Inflammation is associated with insulin resistance and COVID-19 is associated with a cytokine storm. In hospitalized COVID-19 patients, stress hyperglycemia is associated with worse clinical outcomes and is independently related to levels of cytokines that might impair glucose homeostasis; patients with more severe stress hyperglycemia (stress hyperglycemia ratio > 1.14) have higher levels of Interleuin-10 (IL-10) and CXC motif ligand 10 (CXCL10), a higher IL-10/TNF-α ratio, and have been independently associated with severe stress hyperglycemia [122]. Other proposed mechanisms include elevations in angiotensin II that result from a decrease in ACEII levels, T cell imbalance, interference with dipeptidyl peptidase-4 activity, and downstream effects of the RE1-Silencing Transcription factor (REST) [115,123,124,125]. Infection with SARS-CoV-2 resulted in impaired insulin/insulin growth factor signaling pathway genes, including IRS, PI3K, AKT, mTOR, and MAPK in several key tissues, such as lung, liver, adipose tissue, and pancreas [126]. Metformin, a medication used in the treatment of DM that can alter each of these four mechanisms, is being investigated in the treatment of COVID-19 [127].

Increasing evidence is consistent with the ability of SARS-CoV-2 to cross the BBB [128]. Certainly, it can infect the brain endothelial cells which comprise the BBB [129] and the S1 protein, the viral attachment protein for SARS-CoV-2, is able to cross the BBB [130]. BBB dysregulation occurs in COVID-19, as would be expected secondary to the cytokine storm [128,130]. Although the possibility exists for interactions between DM and SARS-CoV-2 in their effects on the BBB, no such studies appear to have been conducted to date.

## 3. Simultaneous Exposure to More Than One Virus

The winter of 2022–2023 has been labeled as a tridemic as influenza, RSV, and COVID-19 are all of concern during this flu season. As such, infection by more than one virus is possible. For example, coinfection of RSV with COVID-19 produces hybrid viral particles that might affect virus pathogenesis and enable immune evasion [131]. Differences in viral kinetics might be important in effects of combined viral exposures. The RSV viral titer increases at a slower rate and reaches its peak value later than the influenza virus does [132]. There is an ongoing discussion about whether combined viral exposures of influenza, RSV, and COVID-19 have additive, synergistic, or interference effects [133]. Additive, synergistic, and interference effects might involve interferon and other cytokine signaling [134].

## 4. Conclusions and Future Perspectives

Several viruses can affect insulin sensitivity and blood–brain barrier (BBB) function. In addition to endothelial cells, other cells of the neurovascular unit including pericytes and astrocytes can also be affected by viruses. With world-wide travel, shipments of animals and animal products, and the hunting and trading of wild animals, there is an increased concern for how some viruses might negatively affect human health. As exposure to more than a single virus at the time is becoming more common, increased efforts are warranted to determine how combined viral exposures affect insulin sensitivity and BBB function.

## Data Availability

Not applicable.
